# Neurological Soft Signs in Aging, Mild Cognitive Impairment, and Alzheimer’s Disease – The Impact of Cognitive Decline and Cognitive Reserve

**DOI:** 10.3389/fpsyt.2015.00012

**Published:** 2015-02-11

**Authors:** Nadja Urbanowitsch, Christina Degen, Pablo Toro, Johannes Schröder

**Affiliations:** ^1^Section of Geriatric Psychiatry, University of Heidelberg, Heidelberg, Germany; ^2^Institute of Gerontology, University of Heidelberg, Heidelberg, Germany; ^3^Department of Psychiatry, Centro Interdisciplinario de Neurociencias, School of Medicine, Pontificia Universidad Católica de Chile, Santiago, Chile

**Keywords:** NSS, MCI, AD, cognitive reserve, ILSE

## Abstract

**Objectives:** Neurological soft signs (NSS), i.e., minor motor and sensory changes, are a common feature in severe psychiatric disorders. We sought to establish the frequency of NSS in patients with mild cognitive impairment (MCI) and Alzheimer’s disease (AD) on basis of a large population-based sample and to identify their neuropsychological correlates including cognitive reserve.

**Methods:** Neurological soft signs were examined using an abbreviated version of the Heidelberg NSS Scale in 221 “old” participants born between 1930 and 1932 (63 with MCI, 15 with AD, 143 healthy old controls) and 256 healthy “young” participants (born between 1950 and 1952) of the population-based interdisciplinary longitudinal study of aging. Subjects received thorough neuropsychological testing; years of school education were used as a proxy for cognitive reserve.

**Results:** Neurological soft signs scores were significantly (*p* < 0.001) higher in the AD patients (5.6 ± 3.11) than in the healthy old controls (2.8 ± 1.90) and in the MCI patients (3.0 ± 1.96). This result was confirmed after years of school education, which were inversely correlated (*r* = −0.25; *p* < 0.001) with NSS were entered as a covariate. In the patients, but not in the controls, NSS were significantly correlated with deficits in executive functioning and visuospatial functioning. Comparison of NSS scores between “old” (2.84 ± 1.9) and “young” (2.46 ± 1.97) controls yielded only minor, non-significant differences after education (13.86 ± 3.0 vs. 14.61 ± 2.48 years, respectively) was controlled for.

**Conclusion:** Our results demonstrate that NSS are frequently found in mild AD, but not in MCI. NSS refer to frontal-executive deficits and visuospatial dysfunction rather than age *per se* and can be partly compensated for by cognitive reserve.

## Introduction

Recent studies (1, 2) demonstrate that neurological soft signs (NSS) – i.e., minor motor and sensory deficits – are frequently found in Alzheimer’s disease (AD) and mild cognitive impairment (MCI) as they were consistently described in other major psychiatric conditions such as bipolar disorder or especially schizophrenia (3, 4). Corresponding to the significant association found between NSS and negative symptoms in schizophrenia (5) and Seidl et al. (2) reported a significant correlation between NSS and apathy in a large group of nursing home residents (*n* = 120) with mild to severe AD. Similar to earlier reports in schizophrenia [reviewed in Ref. (6)], Seidl et al. (2) did not find NSS to be associated with neuroleptic treatment. However, other important aspects of NSS known from studies in schizophrenia – in particular, the associations of NSS with neuropsychological deficits (7–10) – were not yet tested in AD. This question is of particular importance as positive effects of physical activity on cognitive performance are generally accepted (11).

It is generally accepted that high levels of physical activity are associated with a reduced risk for developing AD potentially by facilitating the brain and cognitive reserve. Another study of our group (11) investigated muscular strength as measured by a vigorimeter and motor coordination, which was operationalized by means of the one-foot-standing test, with respect to the risk of developing MCI or AD in the interdisciplinary longitudinal study of aging (ILSE). In the framework of this prospective population-based study, subjects (born 1930–1932) were examined at three examination waves (*t*_1_:1993/1994; *t*_2_: 1997/1998; *t*_3_: 2005/2007). The study demonstrated that motor coordination evaluated at *t*_1_ but not muscular strength was a significant predictor of cognitive impairment at *t*_3_.

Another important question refers to the nature of NSS as signs of generalized rather than discrete cerebral changes. Along with this, neuroimaging studies in schizophrenia identified a number of dispersed cerebral sites associated with NSS (12–15). Hence, one may expect to find increased NSS scores in manifest AD where cerebral changes involve large parts of the brain rather than in MCI where cerebral changes still remain localized in the medial temporal lobe. Accordingly, NSS scores should be rather stable in the process of healthy aging since the latter does not involve major cerebral changes.

We therefore thought to investigate NSS with respect to neuropsychological deficits and school education, as a proxy of cognitive reserve and to compare NSS scores between patients with MCI or AD and healthy old controls. In addition, NSS scores obtained in the latter were compared with scores measured in young controls to identify age-related changes.

## Materials and Methods

### Participants

The ILSE-study is based on two birth cohorts born during 1930–1932 and 1950–1952 who were randomly recruited according to community registers in the urban regions of Leipzig (Saxony) and Heidelberg/Mannheim (Palatine). The study was approved by the ethical committee of the University of Heidelberg and written informed consent after complete description of the study to the subjects was obtained. The first examinations took place between December 1993 and January 1996. Data of the present study correspond to the third examination wave, which were performed between 2005 and 2008, i.e., more than 12 years after the initiation of the study. At this time, 381 participants of the 1930–1932 cohort and 408 participants of the 1950–1952 – corresponding to more than 75 and 80%, respectively, of the original cohorts – could be reinvestigated. Participants with a current episode of major depression, substance abuse, anxiety disorder, bipolar disorder, schizophrenia, or meeting ICD 10 criteria for a cognitive disorder due to a general medical condition were excluded from the analysis since these conditions usually course with both neurological signs and cognitive deficits and may overlap with dementia. Hence, a sample of 477 participants was available for analyses comprising 256 participants of the 1950–1952 cohort and 221 participants of the 1930–1932 cohort.

Sixty-three participants out of 221 (28.51%) were diagnosed with MCI and 15 with AD (6.79%) in the 1930–1932 cohort. Because we were interested in investigating the effects of age on NSS performance 256 otherwise healthy participants from the young cohort were also included. Hence, three groups were constituted: healthy young controls, (*n* = 256), healthy old controls (*n* = 143) old-aged with MCI (MCI; *n* = 63), and old-aged with AD (AD; *n* = 15). All participants selected for the present study had complete core data sets (socio-demographic variables, diagnoses, NSS), while the thorough neuropsychological test battery could be completed in the whole group. The mini-mental state examination [MMSE, (16)] was not applied in the young cohort.

### Survey measures

All participants were carefully screened for physical and mental health by extensive clinical interviews, physical examinations, laboratory tests, and a thorough assessment of neuropsychological functioning. Cognitive assessment included the MMSE and subtests of the Nürnberger-Alters-Inventar [NAI, (17)] and the Leistungsprüfsystem (18), both of which are well-established and commonly used test batteries in Germany [for more details, see Ref. (19)]. Subjective cognitive complaints were assessed by interviewing and applying the respective items of the Nürnberger Selbsteinschätzungsliste [NSL; (20)].

Neurological soft signs were examined by using the modified version (2) of the Heidelberg Neurological Soft Signs Scale (4), which included five items, named – (a) finger-to-nose movement, (b) diadochokinesia, (c) pronation and supination, (d) finger-thumb opposition, and (e) mirror movements. The scores of the items were scaled from 0 (no prevalence) to 3 (marked prevalence), with a total score of maximum 18. The subtests were selected from the original scale based upon clinical and research experience collected with demented patients. Examination subtest had to be easy to understand by AD patients.

### Psychiatric diagnoses

Clinical axis I diagnoses were obtained by using the German version of the Structured Clinical Interview for the DSM-III-R [SKID I, (21)]. MCI was defined by using the aging-associated cognitive decline (AACD) criteria, which were already applied in the first two examination waves (19, 22). Diagnostic criteria for AACD have been proposed by the international psychogeriatric association (23) and include (1) subjective impairment: a report by the individual (or a reliable informant) that cognitive function has declined and (2) objective impairment in any of the following cognitive domains, as indicated by a neuropsychological test performance of at least 1 SD below normal age and educational levels: memory and learning, attention and concentration, abstract thinking (problem solving, abstraction), language, and visuospatial functioning. AD was diagnosed according to the NINCDS–ADRA (24). All diagnoses were confirmed by both specialists in old age psychiatry involved.

### Statistical analyses

Clinical variables, NSS, and neuropsychological performances were compared between groups by calculating separate analyses of covariance (ANCOVA) with group (healthy young controls vs. healthy old controls vs. MCI vs. AD) as predictor variable and years of education as covariate. *Post hoc* comparison analyses were based on Duncan’s test. The potential relations between NSS, clinical variables, and neuropsychological findings were addressed by calculating Pearson product-moment correlation coefficients, which were controlled for years of education. All computations were performed by using the 9.2 version of the SAS Software.

## Results

**Figure 1:**
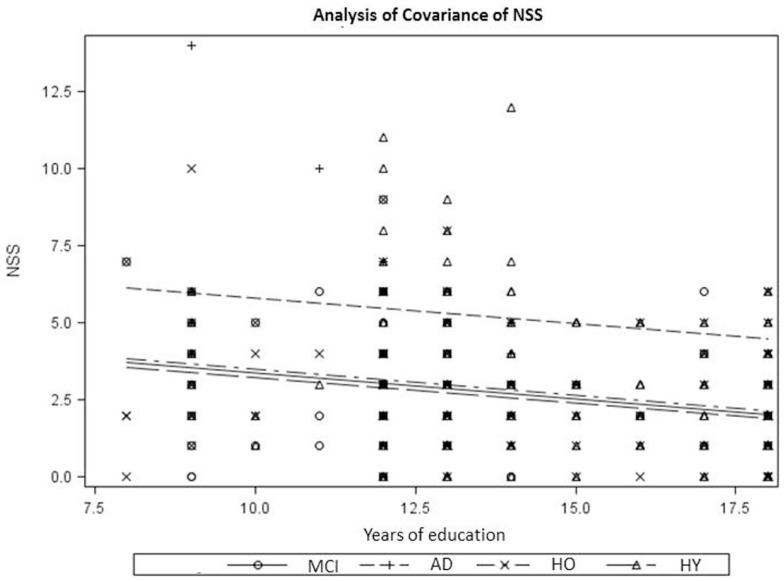
**NSS and years of education across diagnostic subgroups**. NSS, neurological soft signs; HO, healthy old controls; HY, healthy young controls; MCI, patients with MCI; AD, patients with Alzheimer’s disease.

The clinical characteristics, neuropsychological performance, and total NSS scores obtained in the diagnostic groups are summarized in Table 1. Diagnostic groups differed significantly with respect to age and years of school education but showed no significant differences in sex distribution. As expected, AD patients scored significantly lower than MCI patients, followed by healthy old controls, on the MMSE. With respect to neuropsychological test performance, three patterns of group differences arose: performance on verbal memory, digit symbol, D2, and visuospatial thinking tests conformed to the expected order with the highest scores achieved by healthy young controls, followed by healthy old controls and MCI, and the lowest scores obtained by AD. In the subtests, verbal memory recognition, verbal fluency, and finding similarities, significant group differences were found only between MCI and AD, while healthy young controls and healthy old controls showed similar results. AD patients demonstrated significantly more NSS compared to the other three subgroups. As demonstrated in Figure 1 across all diagnostic groups, NSS scores were inversely correlated with years of school education (*r* = −0.25; *p* < 0.001). The ANCOVA with years of school education as a covariate [*F*(4,472) = 15.78, *p* < 0.0001] yielded a significant main effect (type III ss) of diagnostic group on NSS scores [*F*(3,473) = 7.95, *p* < 0.0001].

**Table 1:** **Demographic and neuropsychological characteristics by subgroup with the results of a Duncan test at the 5%-level**.

	Healthy young controls (*n* = 256)	Healthy old controls (*n* = 143)	MCI (*n* = 63)	AD (*n* = 15)	*F* (df)	*p*	Duncan/Chi Square
**DEMOGRAPHICS**							
Age	55.15 (0.97)	73.94 (0.99)	74.21 (1.03)	74.73 (1.03)	14525 (3, 473)	<0.001	HY < HO = OMCI = OAD
Sex (women/men)	126/130	75/68	32/31	7/8	n.a.	n.s.	Chi-Sq: 0.469
Education	14.61 (2.48)	13.86 (2.99)	12.22 (2.41)	11.20 (1.78)	20.07 (3, 473)	<0.001	HY = HO > OMCI = OAD
MMSE (*n* = 218)	n.a.	28.91 (1.12)	28.07 (1.41)	24.13 (2.39)	90.02 (2, 215)	<0.001	HO > OMCI > OAD
**NEUROPSYCHOLOGY**							
Verbal memory: immediate recall (*n* = 438)	14.59 (3.16)	12.36 (3.47)	10.35 (3.46)	7.5 (3.50)	31.7 (3, 434)	<0.001	HY > HO > OMCI > OAD
Verbal memory: delayed recognition (*n* = 438)	7.93 (2.27)	6.91 (2.35)	5.81 (2.50)	4.0 (2.63)	14.5 (3, 434)	<0.001	HY = HO > OMCI > OAD
DST (*n* = 438)	53.94 (9.33)	44.15 (9.51)	36.11 (8.73)	25.8 (8.94)	72.4 (3, 434)	<0.001	HY > HO > OMCI > OAD
D2 (*n* = 432)	164.45 (32.06)	136.79 (33.08)	105.67 (33.93)	76.67 (43.37)	50.1 (3, 428)	<0.001	HY > HO > OMCI > OAD
Finding similarities (*n* = 439)	26.64 (4.17)	26.77 (4.07)	22.6 (6.27)	16.0 (6.62)	18.1 (3, 435)	<0.001	HY = HO > OMCI > OAD
Verbal fluency (*n* = 439)	32.38 (9.36)	32.15 (8.23)	24.47 (8.48)	18.8 (8.9)	11.0 (3, 435)	<0.001	HY = HO > OMCI > OAD
Visuospatial thinking (*n* = 437)	25.41 (6.04)	21.79 (5.72)	17.53 (7.41)	11.56 (6.93)	24.2 (3, 433)	<0.001	HY > HO > OMCI > OAD
NSS	2.46 (1.97)	2.84 (1.91)	3.0 (1.96)	5.6 (3.11)	8.0 (3, 473)	<0.001	HY = HO = OMCI < OAD

*MCI, mild cognitive impairment; AD, Alzheimer’s disease; MMSE, mini-mental state examination; DST, digit symbol test; D2, D2-test; NSS, neurological soft signs; n.a., not applicable; n.s., not significant*.

To further analyze the correlations between NSS and neuropsychology deficits, the subgroups of patients with MCI and AD were collapsed. Within this joint group of cognitively impaired, NSS scores were significantly inversely correlated with scores on the MMSE, on the DST, and on the tests for visuospatial functioning and verbal fluency, processing speed, and cognitive flexibility, respectively (Table 2).

**Table 2:** **Neuropsychological correlates of NSS in patients with MCI or AD**.

NP-test	MMSE	Verbal memory (immediate recall)	Verbal memory (delayed recognition)	DST	D2-test	Finding similarities	Verbal fluency	Visuospatial thinking
**NSS**
*r* uncorrected (corrected)	*r* = −0.45**	*r* = −0.08(*r* = −0.04)	*r* = −0.03(*r* = −0.03)	*r* = −0.37* (*r* = −0.39*)	*r* = −0.13 (*r* = −0.15)	*r* = −0.21 (*r* = −0.18)	*r* = −0.37* (*r* = −0.37*)	*r* = −0.41** (*r* = −0.36*)

*Values corrected for education in brackets. Bold font indicates significant correlations*.

*NP, neuropsychology; MCI, mild cognitive impairment; AD, Alzheimer’s disease; MMSE, mini-mental state examination; DST, digit symbol test; D2, D2-test; NSS, neurological soft signs*.

*p < 0,005; **p < 0.001.

## Discussion

The main findings of the present study can be summarized as follows: significantly increased NSS scores (i) were found in patients with AD but not in patients with MCI or healthy old controls compared to healthy young controls, (ii) correspond to deficient executive functioning and visuospatial apraxia and can be partially compensated for by cognitive reserve, and (iii), are not the sequelae of aging *per se*.

The present study confirmed increased NSS scores in patients with mild AD as previously described by a number of studies (2, 25, 26), which also examined patients in more advanced stages of the disease. This increase was not observed patients with MCI. In contrast, Li et al. (1) reported significantly increased NSS scores – which particularly involved motor coordination signs – in 29 MCI patients who were compared with 28 healthy controls. This discrepancy might refer to methodological differences, such as sample size and mode of recruitment, as well as the relatively greater severity of cognitive deficits in their MCI group, in particular (mean MMSE scores: 26.07 ± 2.33 vs. 28.07 ± 1.41, respectively).

Similar to the study of Chan et al. (27), NSS scores were significantly correlated with neuropsychological deficits involving executive functioning (verbal fluency, digit symbol test) and visuospatial thinking. Verbal memory was not associated with NSS. In the light of these findings, the significant correlation of NSS and logical memory reported by Chan et al. (27) may refer to the fact that the latter also involves some aspects of executive functioning in the sense of mnestic strategies. It is notable that similar associations between NSS and executive deficits were also reported in schizophrenia [for review see Ref. (7)] and in HIV associated neurocognitive disorders (28). That motor performance in AD is related to executive functioning is further supported by the results of dual-task studies, which even yielded interactions between fall risk and executive dysfunctions (29–32).

In all subgroups under investigation, NSS were inversely related with years of school education as a marker of cognitive reserve. This association corresponded to a wealth of studies [for review see Ref. (11)], which identified motor abilities to be a protective factor for cognitive decline and could be mediated by the beneficial effects of aerobic exercise on the hippocampus (33) and the frontal cortices (34).

Despite the large sample size, a comparison of NSS scores between healthy young controls and healthy old controls yielded only minor, non-significant differences. In contrast, Chan et al. (27) who examined 180 subjects aged 60–96 suggested that NSS were very common among the elderly. Their study, however, differs with respect to a variety of important methodological details from the present investigation, among them the definition of cognitively intact controls by a MMSE score of >24. This definition may well have led to the inclusion of patients with mild AD who often showed MMSE scores up to 26. Along with this, educational levels were in the range of 6.8 ± 4.4 years and do not compare with the respective value found in our sample. Similarly, Kodama et al. (35) found deficits in diadochokinesis, finger-to-nose test, and tandem gate to increase with age in 348 subjects aged 60–89. However, educational level and MMSE scores were considerably lower than in our birth cohorts rendering a direct comparison of the results difficult. Therefore, our finding only applies to the rather “young” old investigated here and needs to be confirmed in the further course of the ILSE when subjects have reached a higher age.

Another important methodological limitation of our study involves the fact that only motor NSS were examined. Motor NSS are generally considered to be of particular importance (5) and can be easily applied (2). Likewise, Kodama et al. (35) demonstrated significant age effects only for the motor NSS cited above and vibration sense of the lower limbs but not for other sensory NSS.

As hypothesized, our investigation of two large birth cohorts confirmed increased NSS scores in patients with mild AD but not patients with MCI. According to our findings, NSS do not appear to be the sequelae of healthy aging since the two birth cohorts examined showed only minor, non-significant differences with respect to NSS. That NSS were found to be significantly associated with executive dysfunction corresponds to earlier studies in patients with schizophrenia. However, NSS are not only a marker of brain damage in AD but also reflect aspects of cognitive reserve. Longitudinal studies are necessary to establish the stability of NSS in aging, their prognostic value in MCI and – ultimately – to determine norm values for NSS. From a clinical standpoint, NSS should be considered as a reliable and easy to administer tool in the routine examination of patients with cognitive decline.

## Conflict of Interest Statement

The authors declare that the research was conducted in the absence of any commercial or financial relationships that could be construed as a potential conflict of interest.
